# The domestic violence fatality review clearinghouse: introduction to a new National Data System with a focus on firearms

**DOI:** 10.1186/s40621-019-0182-2

**Published:** 2019-02-25

**Authors:** Neil Websdale, Kathleen Ferraro, Steven D. Barger

**Affiliations:** 1 Family Violence Institute Northern Arizona University, PO Box 1500, Flagstaff, AZ 86011 USA; 2 Department of Sociology, Family Violence Institute Northern Arizona University, PO Box 1500, Flagstaff, AZ 86011 USA; 3 Department of Psychological Sciences, Northern Arizona University, PO Box 15106, Flagstaff, AZ 86011 USA

**Keywords:** Intimate partner violence, Homicide, Epidemiology, Firearms, Injury prevention

## Abstract

**Background:**

In the US more than 1 in 4 women and 1 in 7 men have experienced severe physical violence by an intimate partner. The most severe violence, violence that ends in death, disproportionately affects women. Current or former male intimate partners commit the majority of homicides of females and fifty to 60 % of these homicides are perpetrated with firearms. Most murder-suicides involve intimate partners and the vast majority of these cases are women murdered by intimate partners using a firearm. Little data exist to illuminate the social and legal circumstances surrounding firearm use in intimate partner homicide. Here we describe US Domestic Violence Fatality Review Teams and the planning and development of a National Clearinghouse for Domestic Violence Fatality Reviews. Among other things, the National Clearinghouse will centrally record and harmonize reviews across the US through standardized reporting templates and protocols for gathering de-identified intimate partner homicide case information.

**Conclusion:**

Domestic violence fatality reviews provide a promising yet underutilized data source to understand the links between firearms and domestic violence related deaths. The nascent Clearinghouse can inform policy approaches to address intimate partner homicide as well as firearm-related violence in the United States.

In the US more than 1 in 4 women and 1 in 7 men have experienced severe physical violence by an intimate partner, e.g. being hit, burned, choked, or assaulted with a weapon (Black et al., 2011). The most severe violence, violence that ends in death, disproportionately affects women. In 2015 64% of all female homicides were perpetrated by an intimate partner and 93% of all women killed were killed by someone they knew (Violence Policy Center, 2017). Firearms figure prominently in these killings. Fifty to 60 % of male perpetrators of intimate partner homicide kill with firearms (National Archive of Criminal Justice Data, 2015; Petrosky et al., 2017). Most murder-suicides involve intimate partners (72%) and the vast majority of these cases are women murdered by intimate partners using a firearm (Violence Policy Center, 2015). Policy approaches, such as laws requiring intimate partner violence (IPV) offenders to surrender firearms, are associated with lower rates of intimate partner homicides (Diez et al., 2017; Hemenway, 2017; Lee et al., 2017; Loftin et al., 1991). However, more and better data regarding the social and legal circumstances surrounding firearm use in intimate partner homicide is essential to understand firearm deaths and to protect potential victims (Dzau & Leshner, 2018; Rand Corporation, 2018). Here we describe extant US Domestic Violence Fatality Review Teams and the planning and development of a National Clearinghouse for Domestic Violence Fatality Reviews (hereafter “the Clearinghouse”). The Clearinghouse creates a registry of information about teams and the cases they review, builds communications among teams, and raises the possibility of identifying issues not readily apparent at the local/state level (e.g. the relationship between the availability of emergency medical communications/responses and regional rates of intimate partner homicide). Because Domestic Violence Fatality Review Teams include firearm use as part of their detailed analysis of intimate partner homicides, the Clearinghouse represents an emerging opportunity to advance policy and practice specific to firearms and more broadly to the prevention of intimate partner homicide.

## Introduction to domestic violence fatality review teams

Domestic Violence Fatality Review Teams (hereafter “review teams”) identify homicides, suicides, and other deaths caused by, related to, or somehow traceable to domestic violence and review them to develop preventive interventions (Dawson, 2017; Websdale, 2010; Websdale, 2012; Websdale et al., 2017). Domestic violence fatality reviews emerged in the early 1990s and took at least three forms. One form was informal courthouse groups which examined the internal workings of prosecutorial and court practices to identify possible prevention measures (e.g. in Honolulu, HI, and Washoe County, NV; Websdale et al., 2001; Websdale et al., 1999). A second form involved ad hoc public commissions analyzing one case in meticulous detail (Websdale et al., 2001). A third form comprised less detailed reviews of killings within particular states or jurisdictions. The latter tended to highlight aggregate case characteristics (e.g. demographic themes) as in a description of 51 domestic violence homicides in Santa Clara County, California (Websdale, 2003) (pp. 26–31).

The goal of domestic violence fatality reviews is to transform the way agencies and stakeholders understand and respond to domestic violence. This transformation occurs through improved communication, coordination and collaboration in the handling of domestic violence cases. This process extends across multiple levels, including the community, social service providers, law enforcement and the legal system (Websdale, 2003; Websdale et al., 1999).

Review teams routinely conduct distinct inquiries into deaths above and beyond any criminal investigation. These include review of police records, autopsies, court documents, medical records and, where possible, review teams conduct de novo interviews with persons knowledgeable about the case and the history of those involved, such as family, friends, neighbors, service providers and in some cases perpetrators. These interviews often reveal details that are not captured by administrative criminal justice records, e.g. incarcerations in other jurisdictions, histories of abuse and trauma. In contrast to the criminal investigation, which is directed at criminal responsibility and potential prosecution, review teams develop a broad understanding of the case in order to identify potential preventive interventions. To facilitate this process reviews nearly always occur after the homicide case has been fully adjudicated.

Reviews are conducted using a “subculture of safety” philosophy, emphasizing risk management and continuous improvement rather than fault-finding. This emphasis parallels prevention approaches in other high-hazard domains such as aviation (Fielding et al., 2010), medicine (Huckman & Raman, 2015) and nuclear power (International Atomic Energy Agency, 2002). Teams receive technical assistance from the National Domestic Violence Fatality Review Initiative (NDVFRI; ndvfri.org) which has been supporting teams since 1997.

**Fig. 1:**
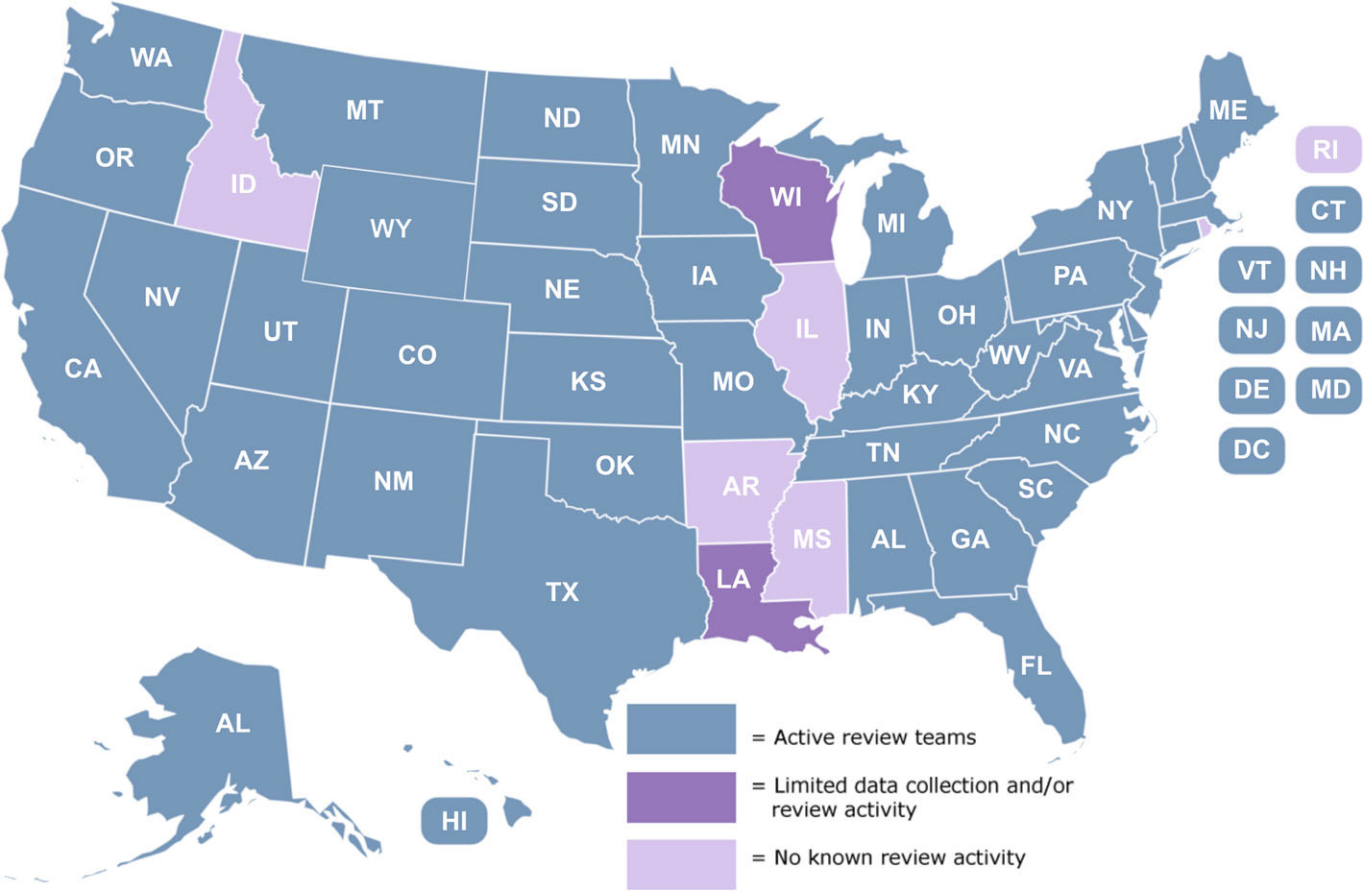
Map of US Domestic Violence Fatality Review Team Activity

**Fig. 2:**
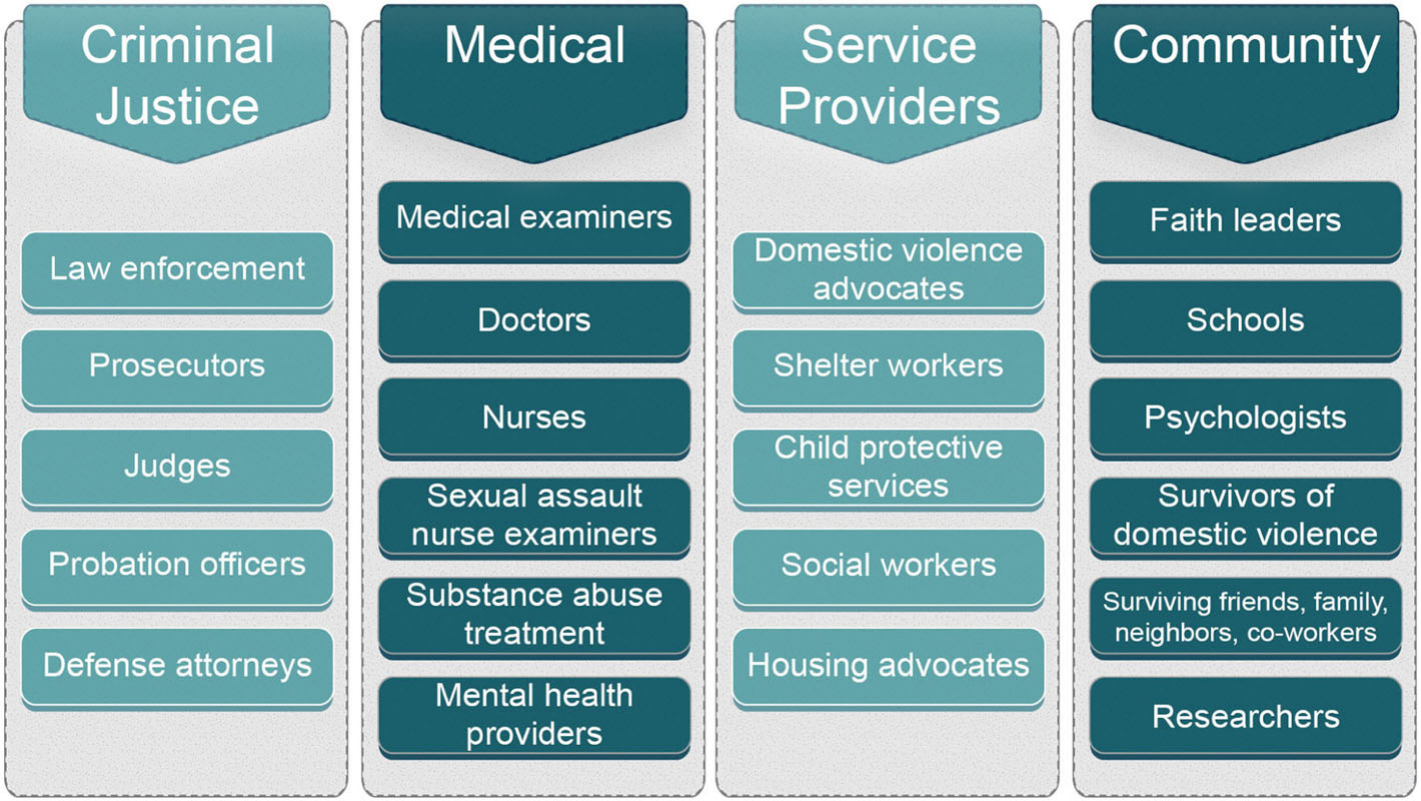
Membership representation of domestic violence fatality review teams

Approximately 200 teams operate in 45 states (Fig. 1). Most were established by legislation and operate at local, regional and state levels. Florida, for example, has 25 county teams as well as a statewide team. Review team representation is defined by statute (Florida Statutes Section 741.316). Arizona has teams representing east and west portions of Maricopa County, a City of Phoenix team, and 10 other county teams; Montana operates a statewide and an Indian Country team. By design, review team membership is diverse and can include medical examiners, physicians, nurses, victim service professionals, public health professionals as well as local law enforcement and citizens at large (Fig. 2). Thus, review teams embody the interprofessional collaboration advocated to address critical public health issues (Butkus et al., 2018; Weiner et al., 2007).

Child death reviews are similarly prevention oriented but have a longer history and encompass all causes of child death rather than those specifically related to family violence, e.g. crib deaths, unintentional injuries (Durfee et al., 1992). In the US there are approximately four and a half times more unintentional injury deaths than homicides among US children ages 1–14 (Xu et al., 2018) and this larger prevalence necessarily results in less detailed death reviews, with perhaps as many as a half dozen reviews completed in an hour. These reviews tend not to identify the detailed case dynamics found in adult intimate partner violence reviews, but have led to innovative policy recommendations such as securing pool areas with fences to reduce drowning (Centers for Disease Control and Prevention & National Center for Injury Prevention and Control, 2012; National Center for Fatality Review and Prevention, 2016). In contrast, domestic violence reviews, some of which can extend over a full day or more, examine mostly homicides and investigate in depth the trajectory of intimate partner violence across the course of the relationship.

### Case types and information collected in fatality reviews

Case types include, but are not limited to, intimate partner homicides, homicide-suicides, familicides, family killing (deaths involving non-intimate partners, e.g. children killing parents), near deaths, sexual competitor killing (e.g. former boyfriend killing new boyfriend), suicide and bystander deaths (e.g. co-worker deaths in a domestic violence-related workplace killing). Teams do not routinely review the deaths of police officers killed in the line of duty handling domestic violence cases. Neither do they routinely review cases where officers have killed a perpetrator or victim of domestic violence. Indeed, the Arizona domestic violence fatality review statute expressly prohibits teams reviewing fatal incidents of domestic violence “committed by an on-duty police officer acting within the scope of employment” (Arizona Revised Statute, 41–198 K2). Teams have rarely reviewed cases where a police officer was the victim or perpetrator of an intimate partner homicide. In such instances, teams deploy their own definitions of domestic violence related deaths.

Although teams review a variety of case types, intimate partner homicide is the most common. Teams gather both public and confidential information which can include the location and circumstances of the homicide, the victim’s relationship status, histories of trauma for victim and perpetrator, alcohol or other drug involvement and the type of weapon, if any, used in the homicide. Teams develop a chronology of events, establishing a timeline of how cases flow through systems (law enforcement, social services, judicial), the nature of any agency and stakeholder involvement, how agency and stakeholder involvement is shaped by bureaucratic practices and policies, and the role of human and organizational factors (Robertson et al., 2016). These elements are largely missing from studies of risk and protective factors for intimate partner violence (Yakubovich et al., 2018) and from other reporting systems such as the National Violent Death Reporting System (see below).

Reviews are also able to document the presence and timing of important firearm-related activities. These include registered firearms sales, mandated waiting periods, surrender/non-surrender of firearms by prohibited possessors, Craigslist (online) purchases and whether or not the intimate partner homicide was committed with a stolen firearm. In addition, risk factors identified in prior reviews, such as strangulation, being threatened with a weapon, recent separation or being beaten while pregnant, are routinely examined. Timelines show that risk markers often intensify in the months and weeks preceding the death, providing insights absent from incident-based investigation. Detailed inquiry is a hallmark of fatality reviews and these reviews are in turn capable of revealing sources of vulnerability and safety as they relate to firearms and other means of lethal violence.

### Case review examples with policy action

The Vermont Domestic Violence Fatality Review Commission illustrates review teams’ work and the concrete preventive actions initiated following review. The Commission operates under the auspices of the Office of the Attorney General in consultation with the Vermont Council on Domestic Violence (Vermont statute 15 VSA Sec. 1140). The Review Commission is tasked with identifying community strengths and weaknesses as they relate to domestic violence prevention and identifying barriers to safety. The Commission also recommends policies, practices and services that will encourage collaboration and reduce domestic violence fatalities. We describe two reviews from the Vermont Commission encompassing 1) the homicide of an intimate partner followed by the suicide of the perpetrator; and 2) the suicide of a long time domestic violence perpetrator.

Mr. Y and Ms. X were living together with Ms. X’s child from a previous relationship. Both Mr. Y and Ms. X were under the supervision of the Vermont Department of Corrections at the time of their deaths and family members described their relationship as “very volatile” (State of Vermont, 2010). Mr. Y had prior felony convictions for domestic violence and had been the subject of multiple relief from abuse orders. He had made serious violent threats against Ms. X and others, had engaged in stalking, and his violent threats towards Ms. X escalated prior to the homicide. Mr. Y was prohibited from possessing firearms under the Federal Gun Control Act yet he still had access to weapons. They had been together 8 months when Mr. Y used a firearm to kill Ms. X and then himself.

**Fig. 3:**
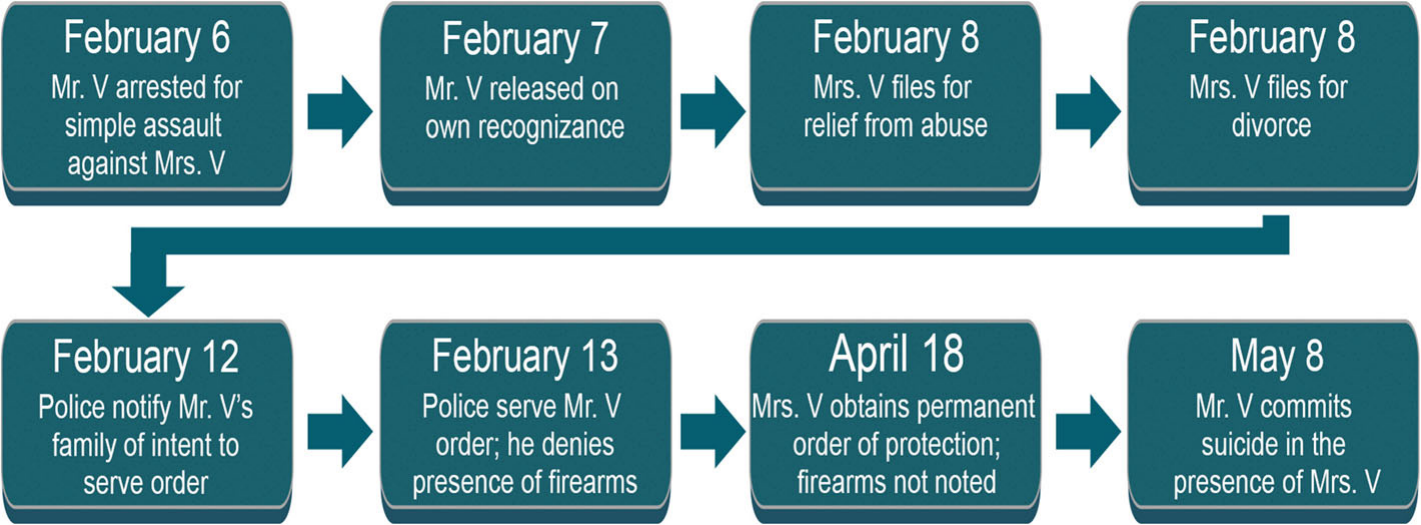
Hypothetical timeline for case excerpts

In the case of an intimate partner suicide, an abused spouse, Mrs. V, filed for divorce alleging “years of physical and sexual abuse” of “intolerable severity” (State of Vermont, 2011). Mrs. V. also obtained a temporary relief from abuse order in which she referenced both Mr. V’s ongoing violence and his possession of four firearms. At that point, Mr. V. was prohibited from possessing firearms because of a prior felony conviction. Although orders of protection are required to be served without advance notice (Vermont statute 15 VSA 1105), in this instance law enforcement had notified Mr. V’s family that officers were looking for him. When eventually served with the temporary order, Mr. V. lied to law enforcement saying he did not have any firearms. At the full protection order hearing, Mrs. V. and her counsel apparently thought Mr. V’s firearms had been removed. Since details about the four firearms did not appear in the permanent order of protection, police had no means of knowing or verifying whether Mr. V. still illegally possessed firearms. These conditions - divorce proceedings, the order of protection, illegal firearm availability and a previous suicide attempt – all suggested elevated risk (see Fig. 3 for a timeline). This timeline ended when Mr. V. took his own life in front of Mrs. V., using one of the firearms documented in the protection order.

As a result of these case reviews, the Vermont Domestic Violence Fatality Review Commission, in collaboration with other concerned coalitions, began the process of strengthening firearms inventory, storage, and relinquishment practices in cases involving prohibited possessors such as Mr. V. The Commission recommended further training for police on service of abuse prevention orders, thus ensuring compliance with Vermont Statute which states that abuse prevention orders “…shall be served at the earliest possible time and shall take precedence over other summonses and orders” (State of Vermont, 2011, p. 10). Specifically, the commission recommended law enforcement officers receive training on the service of court paperwork and that “when serving a protection order, that notice not be provided to the alleged defendant’s family beforehand” (State of Vermont, 2011, p. 11).

In both cases, “there were overlapping issues relating to child support, parental rights and responsibilities, compliance with court orders and domestic violence” (State of Vermont, 2010, p. 16). Consequently, the Commission recommended that the Office of Child Support receive training on the dynamics of domestic and sexual violence and assistance in referring clients to appropriate local services.

As a result of the first case, the Commission noted that the Vermont Department of Corrections was supervising Mr. Y because of a non-violent property crime and neither because of his extensive domestic violence history nor his legal status as a prohibited possessor of firearms. Had his longer-term criminal history been “…reviewed carefully it may have caused Corrections to scrutinize his living arrangements with a fellow DOC [Department of Corrections] supervisee (Ms. X) with a young child” (State of Vermont, 2010, p. 18). Consequently, the Commission adopted a new lethality assessment tool (State of Vermont, 2010) to support more sensitive dangerousness assessments of offenders.

### Limitations of unsystematic reviews and promise of central repository

Despite the potential of review teams’ rich analyses of DV-related deaths, much of the information is in narrative form and often follows unique trajectories shaped by individual case circumstances and local norms. In some states teams describe how firearms were obtained, whether restrictions were imposed, and whether community members were aware of the presence of firearms. Other teams may merely note the type of weapon used in the homicide. Pooling and partially standardizing review team inquiry would leverage the reviews’ capacity for systematic evaluation of intimate partner homicide determinants, particularly with regard to firearms.

Our team, with funding from the US Department of Justice, Office on Violence Against Women (OVW), is developing the Clearinghouse to centrally record and harmonize reviews across the US. Clearinghouse development will be led by the National Domestic Violence Fatality Review team and representatives from volunteer “foundational” review teams from 20 states. The base of foundational teams will grow over time, with ten new volunteer teams added per year, with additional teams joining from the start of year three. By the end of year five, 50 teams will report through the system. These stakeholders will build, extend, and refine standardized reporting templates and reporting protocols for gathering de-identified intimate partner homicide case information. Templates will include a core question set common to all reports as well as more extensive sets of optional queries for teams electing to provide higher resolution case details. In turn, input fields developed in this pilot phase will inform and partially standardize inquiry in subsequent fatality reviews within and beyond the founding review teams. In return for submitting reviews, the Clearinghouse will provide tailored summary descriptions of cases back to teams to improve reports created for communities and governmental bodies. The Clearinghouse concept ultimately seeks to build a permanent national surveillance system for the reporting of data regarding domestic violence deaths.

### Utility of the clearinghouse for data collection and policy

The Clearinghouse has the potential to address gaps in current crime reporting systems. Official data on intimate partner homicide, such as the FBI’s Uniform Crime Reports supplemental homicide reports, are limited by inconsistent and incomplete documentation of intimate partner homicides. For example, in 2015, 47.8% of homicides reported to the FBI did not specify the relationship between the offender and victim (Federal Bureau of Investigation, 2016). Moreover, these systems do not include a category for unmarried former intimate partners and thus would underrepresent intimate partner related violence. The inability of extant national-level data to capture the complex dynamics that precede intimate partner homicide, including corollary firearms data, can in part be addressed by systematic collection of the detailed case reviews of review teams.

Some of the Uniform Crime Reports limitations are addressed in the National Violent Death Reporting System (NVDRS). As of 2018 this system operates in 50 states, Washington, DC and Puerto Rico. Similar to fatality reviews, the NVDRS seeks to better characterize perpetrators and their relationships to victims (https://www.cdc.gov/ncipc/wisqars/NVDRS/About-NVDRS.htm). The NVDRS harmonizes data collection across states and incorporates standard questions on the manner and place of death. Similar information standardization will occur with the Clearinghouse yet the Clearinghouse can incorporate more detailed information relevant to characterizing the homicide. This includes prior criminal and intimate relationship histories, prior threats and abuse, and timing issues such as pending separation or divorce. These elements, along with markers of abuse escalation and severity, may better characterize elevated risk and in turn sharpen prevention approaches. Data on domestic violence service provision and safety planning are other potential Clearinghouse elements that could complement NVDRS reporting. Indeed, in many parts of the country domestic violence specialists also work on NVDRS initiatives. At present fatality review team members in Hawaii, Virginia, and Santa Clara and San Mateo counties (California) are also involved in the NVDRS. More broadly, collaboration between review team members, policy makers, criminal justice authorities, as well as expanding the community of allied professionals and laypersons oriented towards violence prevention, portends a less siloed field and more and higher quality domestic homicide data (Hemenway, 2018).

### Prevention implications of the clearinghouse

Fatality review and the Clearinghouse can contribute to the prevention of intimate partner homicide in a number of ways. From our national field observations, in approximately half of intimate partner homicide cases decedents received no or minimal support services (e.g. one health practitioner consult). This is consistent with work documenting that about half (48%) of the women killed by their intimate partners utilized the criminal justice system for concerns about ongoing intimate partner violence and stalking in the year before their murders (McFarlane et al., 2001). In the other half, decedents received services, often from as many as 10–30 agencies. Among these “receipt of services” cases, teams describe the levels of communication, coordination, and collaboration among service providers as low. Thus, addressing coordinated services across agencies represents a potential opportunity to improve safety.

In an example from rural Santa Cruz County, Arizona, the review team identified courtroom intimidation by the perpetrator and the perpetrator’s family and friends as a major barrier to a victim obtaining a temporary order of protection. In response, the team recommended developing remote sites at health centers where victims can apply for emergency orders of protection in safe spaces. In addition to increased safety, remote requests for protection orders reduce victims’ travel burden for help seeking in remote areas.

### Developing and sustaining review teams

Review work forms a central component of coordinated community responses to domestic violence across the US. It serves a similar role in other liberal democratic states such as Canada, Australia, the United Kingdom, New Zealand, and Portugal. We observe that in the US review activity has occasionally faltered after the departure of one dominant individual review leader or leaders. In contrast, sustainable review activities tend to be perceived as impartial fact-finding initiatives and are comprised of an eclectic array of reviewers with various and, at times, competing perspectives on domestic violence cases. Their reviews tend to be more in-depth and are often supported (but not mandated) by statute. Deeper reviews take longer (a day or two as opposed to an hour or two) but are perceived as more rewarding, engaging to reviewers, and more likely to identify breakdowns in communication, coordination, and collaboration.

### Confidentiality and privacy protections for the clearinghouse

The Clearinghouse development team includes specialist attorneys as well as representatives from the US Department of Justice, Office on Violence Against Women. In addition to following statutory requirements, Clearinghouse activities will adhere to individual team rules, practices, and protocols relevant to confidentiality and privacy. These practices in turn are subsumed under the larger umbrella of US Department of Justice confidentiality guidelines on data acquisition, storage, analysis, and reporting. Data protection specialists will develop mechanisms to securely receive, store, and query de-identified case data as it begins to flow to the Clearinghouse from each of the 20 “foundational” reporting teams. Teams themselves will contribute to every stage of protocol development for data input, structure and storage, consistent with their own state fatality review statutes.

De-identified aggregate data, particularly when embedded in a large database, can mitigate disclosure concerns applicable to both local-level reviews and to states with smaller case numbers. However, information about the role of firearms is not legally protected even though it may be embedded in criminal justice files available to review teams but not readily available to the public. In the Vermont example above, Mr. V’s prohibited possessor status is the kind of information that is not legally protected but is not always revealed in public accounts of homicides. Through such review an individual team might be able to directly plug the “verification” gap in the flow of firearms illustrated by the case of Mr. V. The Clearinghouse will hopefully provide even greater leverage to identify and redress shortcomings in lawful firearm retrieval at state and local levels.

## Conclusion

The Clearinghouse will use systematically gathered review data from an increasingly cohesive group of foundational teams in the service of preventing domestic violence deaths and preserving the safety of domestic violence victims, their families and the community. It is not the primary function of the Clearinghouse to gather data on firearms; rather firearms data are just one necessary domain review teams consider in the broader service of understanding and preventing intimate partner homicides, the majority of which are committed with firearms. Fatality review can be further strengthened by participation from members of the medical and public health communities and others with a shared commitment to protecting public health. Domestic violence fatality reviews provide a promising and underutilized data source to better understand the links between firearms and domestic violence related deaths and the Clearinghouse can more precisely characterize a poorly understood but socially devastating type of firearm-related health burden (Butkus et al., 2018).

## Abbreviations

DV: domestic violence
NDVFRI: National Domestic Violence Fatality Review Initiative
OVW: Office on Violence Against Women
